# *Akkermansia* in the gastrointestinal tract as a modifier of human health

**DOI:** 10.1080/19490976.2024.2406379

**Published:** 2024-09-21

**Authors:** Maria E. Panzetta, Raphael H. Valdivia

**Affiliations:** Department of Integrative Immunobiology, Duke University, Durham, NC, USA

**Keywords:** *Akkermansia muciniphila*, *massiliensis*, *biwaensis*, *ignis*, *durhamii*, microbiota, obesity, diabetes, neurological disease, cancer, immunotherapy, liver, infections, sepsis, asthma, nephropathy, ALS, AD, PD, ASD, MS, helminth, GVHD, IBD, influenza, tuberculosis, *Clostridium difficile*, *Salmonella typhimurium*, epilepsy, NAFLD, CKD, LCMV

## Abstract

*Akkermansia sp* are common members of the human gut microbiota. Multiple reports have emerged linking the abundance of *A. muciniphila* to health benefits and disease risk in humans and animals. This review highlights findings linking *Akkermansia* species in the gastrointestinal (GI) tract to health outcomes across a spectrum of disorders, encompassing those that affect the digestive, respiratory, urinary, and central nervous systems. The mechanism through which *Akkermansia* exerts a beneficial versus a detrimental effect on health is likely dependent on the genetic makeup of the host metabolic capacity and immunomodulatory properties of the strain, the competition or cooperation with other members of the host microbiota, as well as synergy with co-administered therapies.

## Introduction

The microbe *Akkermansia muciniphila* was first isolated two decades ago^1^ and has emerged as an important member of the complex microbial community in the gastrointestinal tract.^2^ Over the last decade, multiple reports have linked the abundance of *A. muciniphila* to health benefits and disease risk.

The genus *Akkermansia* was named after the Dutch microbiologist Dr. Antoon Akkermans, and the species designation *muciniphila* was used to highlight the ability of the first species isolated to thrive using mucin as the sole carbon and energy source. *A. muciniphila* strain MucT represents the first human isolate belonging to the Verrucomicrobiota phylum. This phylum mainly comprises bacteria from marine environments and animal feces. In addition to *muciniphila*, the *Akkermansia* genus includes the species *massiliensis*,^3^ *biwaensis*,^4^ *ignis*^5^ and *durhamii*,^6^ as well as additional potential species within clade AmIII.^7^ The genomes of *A. muciniphila* are ~ 10% smaller than those of other *Akkermansia* species.^6^ Based on metagenomic sampling, approximately half of human fecal samples contain detectable levels of *Akkermansia. A*mong those, *A. muciniphila* is the most common species, representing ~ 70% of all isolates.^6,8^ *Akkermansia muciniphila* is subdivided into subsp *muciniphila* (AMIa) and *communis* (AMIb).^6^ Whether the genetic differences between *Akkermansia* species and subspecies contribute to their impact on human health remains to be determined.

**Figure 1.**
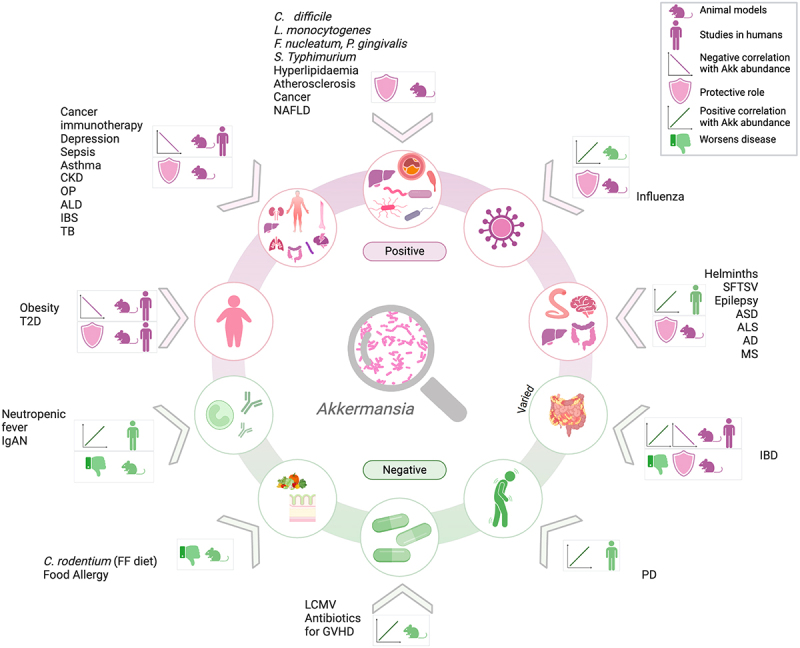
*Akkermansia* orchestrates a spectrum of health outcomes. Graphic summary of the studies reviewed indicating the outcome of correlation (green indicates negative, purple indicates positive) and the intervention studies performed in animal models and/or human cohorts. Acronyms in the figure: CKD, chronic kidney disease; OP, osteoporosis; ALD, alcoholic liver disease; IBS, irritable bowel syndrome; TB, tuberculosis; *C. difficile*, *Clostridioles difficile*; *L. monocytogenes, Listeria monocytogenes*; *F. nucleatum, Fusobacterium nucleatum*; *P. gingivalis, Porphyromonas gingivalis*; *S. typhimurium, Salmonella typhimurium;* NAFLD, nonalcoholic fatty liver disease; SFTSV, severe fever with thrombocytopenia syndrome virus; ASD, autism spectrum disorder; ALS, amyotrophic lateral sclerosis; AD, alzheimer’s disease; MS, multiple sclerosis; IBD, inflammatory bowel disease; PD, parkinson’s disease; LCMV, lymphocytic choriomeningitis virus; GVHD, graft versus host disease; *C. rodentium, Citrobacter rodentium*; FF, fiber free; IgAN, immunoglobulin a nephropathy; T2D, type 2 diabetes.

### The application of animal models to understand the impact of *Akkermansia* on human health

#### Akkermansia *can ameliorate disease in animal models*

Introducing *Akkermansia* into animals can ameliorate disease and modulate the immune system toward an anti-inflammatory response. For instance, repeated administration of *A. muciniphila* MucT has been reported to protect mice from infection by *Clostridioides difficile,*^9^ *Listeria monocytogenes,*^10^ *Fusobacterium nucleatum,*^11^ *Porphyromonas gingivalis*^12,13^ and *Salmonella typhimurium*.^14^ Pasteurized *A. muciniphila* protects against *S. typhimurium* infection,^14^ and the outer membrane protein Amuc_1100 is sufficient to protect against *P. gingivalis* periodontitis.^13^ In the case of *F. nucleatum*, *Akkermansia* can directly block the expression of an *F. nucleatum* virulence factor, thus inhibiting the activation of the NFkB pathway in gingival epithelial cells.^11^ Common mechanisms proposed to explain *Akkermansia* protective role in these infection models include increases in anti-inflammatory responses mediated by IL-10, and expression of tight junction proteins that limit pathogen translocation across epithelial barriers. It is important to consider that these studies focused exclusively on mouse models and involved multiple inoculations of *Akkermansia.*

Mouse models of diseases of impaired lipid homeostasis such as atherosclerosis,^15^ acute and chronic hyperlipidemia,^16^ nonalcoholic fatty liver disease (NAFLD)^17–19^ and nonalcoholic steatohepatitis (NASH)^20^ also point to a beneficial role for *Akkermansia*. Viable *A. muciniphila* MucT was required to diminish inflammation in acute and chronic hyperlipidemia^16^ and atherosclerosis.^15^ For the latter, an increase in the expression of tight junction proteins and reduction in proinflammatory cytokines and macrophage infiltration lead to decreased metabolic endotoxemia-induced inflammation and improved gut barrier.^15^ In a mouse model of hyper triglyceridemia (CREBH-null), administration of *A. muciniphila* increased the expression of LDL receptors, reduced hepatic endoplasmic reticulum stress, and lowered triglycerides, dampening inflammatory responses.^16^ The protection against NAFLD in mouse models involves a decline in hepatic enzymes in the serum,^17,19^ decreased IL-6 in the liver,^17^ reduced expression of NLRP3 and TLR4/NF-kB, and changes in the microbiota composition.^19^ A third study in NAFLD mouse models proposed a synergistic role with *Bifidobacterium bifidum* for activating hepatic farnesol X receptors and suppressing those in the colon, plus increased tight junction protein expression.^18^ The prevention of hepatic inflammation was also reported for a mouse model of NASH, where *Akkermansia* administration decreased TLR2 expression, reduced hepatic M1 macrophages and inflammatory γδT and γδT17 cells.^20^ One of these studies in liver disease proposed that Amuc_1100 was sufficient to mimic the effects of live *Akkermansia*.^19^

Overall, these studies suggest that *Akkermansia* modulation of host responses after repeated administration, especially through dampening of inflammation, can be protective in the context of infection with several pathogens and during hepatic or lipid metabolism-linked diseases. In some studies, viable *Akkermansia* was not required for this effect, with Amuc_1100 being able to mimic the response to viable bacteria in NAFLD.

#### Akkermansia *abundance negatively impacts health in animal models*

In specific contexts, *Akkermansia* can have a detrimental impact on some mouse models of disease. *A. muciniphila* can bloom upon infection with lymphocytic choriomeningitis virus or following antibiotic treatment in an immune-compromised host, e.g., graft versus host disease (GVHD). In the first case, LCMV infection induces anorexia, followed by an increase in *A. muciniphila* abundance, attenuating CD8^+^ T cell response to the virus.^21^ Because *Akkermansia* proliferates robustly in host mucins, its abundance is independent of nutrients consumed by the host, and thus, the relative levels of *Akkermansia* increase during fasting.^21^ Similarly, during hematopoietic stem cell transplantation, *Akkermansia* levels increase as the host’s appetite declines following chemical and radiation therapy. As overall mucolytic activities increase, the intestinal barrier becomes compromised, which increases the risk of neutropenic fevers and GVHD.^22^

The lack of fiber in the diet restricts the growth of glycan-consuming commensals, which cannot rely on mucin and related glycoproteins as the sole carbon and nitrogen source. *Akkermansia* bloom in BALB/c mice fed a fiber-free diet led to thinning of the mucus layer, increased type I and II cytokines, and IgE coating of commensals. This can result in an increased risk of allergic reactions to food allergens.^23^ Moreover, an intervention study in animals on a fiber-free diet with *A. muciniphila* supports the premise that the bacterium’s mucin-degrading capabilities can negatively impact immune health. *A. muciniphila*-induced thinning of the mucus layer increased the vulnerability of mice to *C. rodentium* infections.^24^

Overall, these animal studies suggest that the impact of *Akkermansia* supplementation on its host’s health is modulated by diet and immune status. The complex interactions between hosts, their microbiota, diet, and exposure to microbial pathovars can impact *Akkermansia’s* potential as a probiotic. In addition, understanding the impact of diverse *Akkermansia* species and the proposed mechanism should be considered.

### Akkermansia’s *impact on human health as inferred by clinical associations*

#### *Diseases where* Akkermansia *abundance correlates with positive health outcomes, and Akkermansia can ameliorate disease in animal models and humans*

Since the initial study ascribing a protective role for *A. muciniphila* in metabolic disorders, multiple reports linking *Akkermansia* to positive health outcomes have emerged. The relative abundance of *A. muciniphila* in animal models^25^ and humans^26^ decreases during diabetes or obesity. Moreover, oral administration of *Akkermansia* mitigates disease in animals^25–32^ and has proven beneficial in preliminary clinical studies, improving insulin sensitivity, plasma cholesterol levels, and human inflammation markers.^33^ Foundational studies by DeVos and Cani found that live but not heat-killed *A. muciniphila* can reverse metabolic disorders by increasing endocannabinoids that control inflammation, gut peptide secretion, and gut barrier function in mice.^25^ Since then, pasteurized *A. muciniphila*^27^, *A. muciniphila* Extracellular Vesicles (EVs),^26,27,29^ Amuc_1100,^27,34^ and P9 (a protein capable of inducing glucagon-like peptide-1)^31^ have been shown to be sufficient to improve obesity markers. In vitro studies have suggested that TLR2 is activated by Amuc_1100,^27^ and phosphorylation of the kinase AMPK occurs after exposure to EVs,^26^ hinting at potential mechanisms of action. Mucins may modulate some of these activities, as *A. muciniphila* cultivated in synthetic media, as opposed to mucin-containing media, exhibited greater efficacy in enhancing intestinal barrier function and ameliorating metabolic disorders in mice.^30^

It should be noted that these intervention studies have exclusively concentrated on the *A. muciniphila* strain MucT. There is strong evidence of heterogeneity among *Akkermansia* species and *A. muciniphila* subspecies.^6,35^ How these may diverge in their impact on host physiology is poorly understood.

#### *Diseases where* Akkermansia *abundance correlates with positive health outcomes, and it can ameliorate disease in animal models*

Recent observations indicate that the severity of a broad spectrum of diseases could be potentially impacted by *Akkermansia* abundance. It includes responsiveness to cancer immunotherapies, clinical depression, asthma, sepsis, irritable bowel syndrome, osteoporosis, alcoholic steatohepatitis, chronic kidney disease, and tuberculosis.

In cancer, a revolutionary study demonstrated that a fecal microbiota transplant (FMT) supplemented with *A. muciniphila*, strain CSUR P2261, improved patients’ response to immune checkpoint inhibition (ICI) with anti-PD1 antibodies.^36^ In a mouse model of subcutaneous sarcoma, *A. muciniphila* enhanced IL-12-dependent recruitment of CCR9+CXCR3+CD4+ central memory T cells to tumors and reduced the number of regulatory T cells (Tregs) in the tumor-infiltrating lymphocytes.^36^ Since then, there have been reports of lower *Akkermansia* abundance in patients with non-small cell lung (NSCLC)^37^ and colorectal^38^ cancers. Additionally, *Akkermansia* is enriched in the gut microbiota of patients who respond to immunotherapy in renal cell cancer (RCC) and NSCLC cases.^36,39,40^ However, the level of *Akkermansia* is an important variable as high abundance (>4.66%) is associated with shorter survival as much as with no *Akkermansia*.^39^ In addition, the positive impact on survival is more robust in those colonized with *A. muciniphila* subspecies AmIa (subsp. *muciniphila*).^6^ Positive outcomes require the rest of the microbiota, as indicated by the 50% success rate in tumor response to ICI inhibition in mouse models after FMTs from NSCLC donors supplemented with *Akkermansia*.^39^ In an animal lung cancer model, *A. muciniphila* MucT inhibited tumorigenesis, and it was detected in the blood immediately after gavage and cleared by six hours, suggesting a non-GI mechanism of action in this experimental system.^41^ In colitis-associated colorectal cancer (CAC) animal models, pasteurized *Akkermansia* and Amuc_1100 can delay tumorigenesis by promoting the expansion and activation of cytotoxic T cells (CTLs) in the colon and mesenteric lymph nodes.^42^ Additional anti-tumor mechanisms include the enrichment of M1-like macrophages in the colorectal cancer tumor microenvironment (TME) in an NLRP3-dependent manner.^38^ EVs and Amuc_2172, an acetyltransferase that promotes the secretion of HSP70 in cancer cells, are proposed to promote CD8^+^ T cell activity.^43^ Finally, oral administration of live or pasteurized *A. muciniphila*, or recombinant Amuc_1100, can improve the outcome of IL-2 therapy in B16F10 and CT26 (subcutaneous melanoma and colorectal cancer) tumor-bearing mice. Amuc_1100 increased the CTLs and decreased Tregs in the TME.^44^ In summary, *Akkermansia* can enhance cancer immunotherapies by modulating immune cell infiltration into the TME.

Asthma is a condition in which the inflammation of the airways and an excess of mucus impact lung function. There appears to be a negative correlation between *Akkermansia* abundance in the gut and the severity of asthma in humans.^45,46^ Mouse models of allergen-induced respiratory airway disease support a protective role for *Akkermansia*. The protective effect applies only to pasteurized and live *A. muciniphila*, not heat-killed or supernatants.^45,47^ It is associated with decreased eosinophils in bronchoalveolar lavage (BAL), less secretion of Th2 cytokines in the lung, which are essential for IgE production, and a lymphocyte profile in lung tissues consistent with anti-inflammatory responses.^45^ These findings were supported by reports describing a decrease in airway hyper-responsiveness in lung mast cells, lower house dust mites-specific IgE, suppression of Th2 cytokines and eotaxin in bronchial lavages and increase in cecal SCFA as well as changes in the abundance of SCFA-associated genera in the gut.^47^

The relative levels of *Akkermansia* abundance have been reported to decrease in patients with sepsis and corresponding mouse models of the disease. Oral supplementation of *A. muciniphila* to mouse models of sepsis indicated that live, but not heat-killed, *A. muciniphila* provided protection. A peptide enriched in *Akkermansia* cultures supernatants and in the cecum and plasma of germ-free mice colonized with *A. muciniphila*, Arg-Lys-His (RKH), is responsible for binding TLR 4 and inhibiting systemic inflammation in sepsis mice and piglet models.^48^ *Akkermansia*‘s role in dampening sepsis is yet another example of how this microbe can modulate immunity systemically.

Irritable Bowel Syndrome (IBS) is a prevalent disorder affecting the gut-brain axis, characterized by symptoms such as abdominal pain, bloating, diarrhea, and constipation. In IBS patients who underwent FMT, the engraftment of *Akkermansia* correlated negatively with pain intensity, suggesting a potential role of *Akkermansia* in pain modulation in IBS.^49^ In a separate study involving IBS patients who had not responded to standard treatment for over a year and were treated by FMT, increased levels of *Akkermansia* were observed in individuals who showed lower IBS severity scores.^50^ Consistent with these findings, pasteurized *A. muciniphila* MucT reduced symptoms in a mouse model of colonic hypersensitivity.^51^ In the “Neonatal maternal separation” model, administration of *Akkermansia* led to an increase in ZO-1 expression with reduced epithelial permeability and improved anxiety measurements in a *Citrobacter* infection model. In both cases, a moderate increase in IL-22 expression was observed. Additionally, in vitro capacity was demonstrated to neuromodulate nociceptors in the colon.^51^

Depression stands as the foremost cause of disability globally, with mounting evidence highlighting a role for the microbiota, including studies in germ-free mice.^52^ *Akkermansia* levels were found to be lower in patients with depressive conditions, such as bipolar disorder,^53^ first episode of depression,^54^ and late-life depression.^55^ In contrast, in a cohort that compared severe versus mild depression, *Akkermansia* positively correlated with the severity of the disease.^56^ Similarly, *Akkermansia* was found to be enriched in atypical versus typical depression.^57^ In two studies with mouse models of stress-induced depression, *Akkermansia* was diminished in animals with more severe symptoms.^58,59^ These findings suggest that the association between this microbe and disease status may vary across subtypes of depression. However, these studies await confirmation in larger cohorts as the association between *Akkermansia* abundance with subtypes of depression was derived from a relatively small number of patients, both for atypical depression (15 atypical, 44 typical, 19 controls)^57^ and severity of disease (mild, *n* = 7, moderate, *n* = 18, severe, *n* = 14)^56^ subgroups. Nonetheless, multiple studies in mouse models support the role of *A. muciniphila* in dampening depression.^60–69^ Either *A. muciniphila* MucT,^60,62,65,66,68^ from clade AMIa (subspecies *muciniphila*), or *A. muciniphila* strain GP01,^69^ from clade AMIb (subspecies *communis*),^6^ are sufficient for this effect. Potential mechanisms of action include an increase in serotonin (5-HT) in the circulatory, digestive, and neurological systems; inhibition of serotonin transporter (SERT) expression in the gut, reduction in pro-inflammatory cytokines, upregulation of Brain-derived neurotrophic factor (BDNF); and modulation of the abundance or activities of other members of the microbiota. Live bacteria, EVs,^62^ or Amuc_1100,^61,64,67,68^ have been positively associated with an antidepressant effect. It has been proposed that Amuc_1100 binding to TLR2 increases intestinal serotonin. A truncated form of Amuc_1100 lacking the first 80 amino acids and with a higher affinity for TLR2 can exert antidepressant activity in animals.^64^

The degradation of bone tissue characterizes osteoporosis (OP). Osteopenia denotes a bone mineral density reduction before reaching the OP threshold. *Akkermansia* abundance tends to decrease in the gut microbiota of individuals with osteopenia^70^ and is further reduced in OP.^71^ *Akkermansia* levels are also diminished in Sprague Dawley rats with OP.^72^ Oral administration of *A. muciniphila* MucT to ovariectomized C57BL/6 mice shielded these mice from bone loss.^73^ Both viable *A. muciniphila* and EVs were protective, but pasteurized bacteria were not.^73,74^ The authors proposed that nanovesicles may penetrate and accumulate within bone tissue to exert beneficial effects, but how vesicles would access these sites and inhibit bone loss is unclear.

Patients with alcoholic steatohepatitis (ASH) display lower levels of *Akkermansia*.^75^ In a mouse model of ethanol-induced hepatic injury, administration of *A. muciniphila* prevented and ameliorated steatosis and infiltration of MPO^+^ neutrophils.^75,76^ Because *Akkermansia* was not detected in the hepatic tissue, its protective role was proposed to depend on increased expression of tight junction proteins, with a consequent reduction in endotoxin translocation.^76^

Chronic Kidney Disease (CKD) is a pathological condition characterized by progressive kidney damage, potentially culminating in renal failure. The abundance of *Akkermansia* correlates negatively with CKD.^77^ Supplementation with *Akkermansia* (strain GDMCC 1.1346) improved renal function parameters such as urine protein levels, serum creatinine, and blood urea nitrogen (BUN) serum levels in nephrectomized Sprague-Dawley rats. Investigators observed suppression of epithelial-mesenchymal transition (EMT) and decreased IL1B, IL10, and LPS in circulation.^78^

Lastly, tuberculosis is a bacterial infection caused by *Mycobacterium tuberculosis* that causes severe lung damage. *A. muciniphila* abundance is reduced in patients with active tuberculosis.^79^ Specific type I interferon receptor 1 (encoded by IFNAR1) alleles contribute to an enhanced immune response (higher tumor necrosis factor), decrease levels of *A. muciniphila*, and lead to more severe disease in transgenic mouse models and humans. Oral supplementation of *A. muciniphila* MucT in mouse models of tuberculosis reduces infection, lessens pathology, and reduces circulating TNF. *Akkermansia* exerts its beneficial impact through palmitoleic acid-mediated epigenetic inhibition of TNF.^79^

#### *Diseases where* Akkermansia *abundance correlates with negative health outcomes, but it can lessen disease in animal models*

Several reports suggest that *Akkermansia* is associated with a higher risk or severity of disease in humans. Unexpectedly, in some instances, supplementation with *Akkermansia* is protective in animal models of the same diseases. Examples include infections caused by helminths, influenza, severe fever with thrombocytopenia syndrome virus (SFTSV), and neurological diseases such as Alzheimer’s, Amyotrophic Lateral Sclerosis, Autism Spectrum Disorder, Epilepsy, and Multiple Sclerosis.

Increased *Akkermansia* levels have been reported in humans^80^ and mice^81^ infected with nematodes. However, oral gavage with live or pasteurized *A. muciniphila* MucT in mice infected with the roundworm *Trichinella spiralis* enhanced pathogen clearance via TLR2 independent of type II immunity.^82^ Similarly, infection of mice with the influenza virus leads to an increase in *Akkermansia* levels. But paradoxically, if mice are first gavaged with *A. muciniphila* MucT, the animals display lower pulmonary viral titers, reduced proinflammatory cytokine expression, and enhanced levels of type I and type II interferons upon infection with influenza.^83^ In humans infected with SFTSV, *Akkermansia* levels were higher in patients who survived and inversely correlated with inflammatory markers in serum. In animal models of SFTSV infection, oral gavage with *A. muciniphila* MucT mitigated infection, increasing the percentage of mice survival. *A. muciniphila* produces harmaline, which induces changes in the bile acid profile via BAAT (bile acid-CoA: amino acid N-acyltransferase) expression, thereby offering protection against SFTSV by suppressing NF-KB-mediated systemic inflammation through the transmembrane G-protein coupled receptor-5 (TGR5) pathway.^84^

Alzheimer’s disease (AD) is a progressive neurodegenerative disorder characterized by a decline in cognitive function, memory loss, and behavioral changes. Reports in distinct human populations found increased *Akkermansia* abundance in individuals with AD.^85–88^ However, in a mouse model of AD (APP/PS1), oral administration of *A. muciniphila* GP01 (clade AMIb) improved impaired cognition and anxiety-related behaviors, which paralleled Aβ plaque formation in the cerebral cortex and improved glucose tolerance, intestinal barrier function, and dyslipidemia.^89^ Similar conclusions were made in two different animal models, AD-like Sprague-Dawley rat models injected by AlCl3 and D-galactose,^90^ and AlCl3 exposed zebrafish models.^91^ In both cases, administration of live or pasteurized *A. muciniphila*, respectively, reduced markers of AD. Amyotrophic lateral sclerosis (ALS) is a progressive neurodegenerative disorder that affects nerve cells in the brain and spinal cord. When comparing individuals with ALS to their spouses within one year of diagnosis, *Akkermansia* levels were elevated only in ALS patients.^92^ In mice expressing an ALS-associated human mutated superoxide dismutase 1 (SOD1.G93A), there is a decrease in the *Akkermansia* relative abundance as the disease progresses compared to wild-type littermates. Two *Akkermansia* strains (*muciniphila* MucT and ATCC BAA-2869 (isolated from a squirrel) can improve motor symptoms when given to SOD1.G93A mice. This improvement in ALS correlated with increased nicotinamide levels in the serum and CSF, which could improve mitochondrial function.^93^ Autism Spectrum Disorder (ASD) is a neurodevelopmental disorder characterized by challenges in social interaction, communication, and repetitive behaviors. The association between *Akkermansia* abundance and ASD depends on the population assessed; it decreased in cohorts in Australia^94^ and China^95^ but increased in Ecuador.^96^ The size of the ASD cohorts also varied, which might explain the discrepancies encountered (23 ASD in Australia, 25 ASD in Ecuador, 48 ASD in China). When tested in a mouse model of ASD (C57BL/6J VPA-treated), live *A. muciniphila* MucT activated dopaminergic neurons, improving social deficits.^97^ Cerebral palsy (CP) and epilepsy are two interacting diseases with symptoms that appear in early childhood and denote a disruption in neuronal activity in the brain. *Akkermansia* levels are also increased in children with CP and epilepsy.^98^ An extreme ketogenic diet is an effective treatment for patients who fail to respond to anticonvulsant drugs. In mouse models of epilepsy (6-Hz and *Kcna1*-/-), a ketogenic diet alters the gut microbiota, increases *Akkermansia* relative abundance, and protects against seizures. *A. muciniphila* MucT strain co-administered with *Parabacteroides* confers seizure protection with increased GABA/glutamate in the hippocampus and decreased circulating gamma-glutamyl amino acids.^99^ Multiple Sclerosis (MS) is a chronic autoimmune disease that affects the central nervous system. Earlier studies established a positive correlation between *Akkermansia* abundance and MS diagnosis.^100,101^ In contrast, a later study indicated higher levels of *Akkermansia* were linked to lower disability scores in relapsing-remitting (RRMS) and progressive MS.^102^ It has also been reported that *Akkermansia-*specific IgG increases in the cerebrospinal fluid of RRMS compared to other neurological diseases.^103^ Similarly, *A. muciniphila* significantly increases the differentiation of peripheral blood mononuclear cells into Th1 lymphocytes in vitro, but T lymphocyte differentiation does not occur in multiple lymphoid tissues when administered to germ-free mice.^100^ Subsequent studies ascribed a positive impact of *Akkermansia* on MS symptoms using either *A. muciniphila* MucT or *A. muciniphila* strains (Prog-BWH-J5, RRMS-BWH-H3, Prog-BWH-I7) isolated from patients.^102,104^ A marked surge in *Akkermansia* levels occurs at the peak of the disease in the EAE (experimental autoimmune encephalomyelitis) mouse model and untreated MS patients, which is proposed to be mediated by the microRNA miR-30d. This bloom correlates with an expansion of Tregs, which ameliorates disease in the EAE model.^104^ In addition, *Akkermansia* strain BWH-H3 reduced RORγT+ γδ T cells and IL-17–producing γδ T cells, which are pro-inflammatory.^102^ Interestingly, a comprehensive prospective study in infants who did not develop neurological disorders such as ADHD, ASD, Speech, and intellectual disability in childhood found *Akkermansia* was enriched in healthy children as well as metabolites such as 3,4-dihydroxy-phenyl-propionic acid.^105^ Indeed, considering published observations, it’s plausible to interpret that the increase in *Akkermansia* abundance may represent a response to these neurological disorders rather than being a causative factor.

#### *Diseases where the results are inconsistent for supporting a protective role for* Akkermansia: *the role of genetic diversity and animal models as confounders*

Inflammatory Bowel Disease (IBD) encompasses a group of chronic inflammatory conditions of the digestive tract, represented mainly by Crohn’s disease (CD) and ulcerative colitis (UC). In patients with IBD, some studies report a negative correlation between *A. muciniphila* levels and IBD,^106,107^ while others did not find an association.^108,109^ Evidence indicates that the type of *A. muciniphila* subspecies present may differentially affect protection from CD and UC. Lower levels of subspecies muciniphila (AmIa) are associated with cases of CD and UC, but lower levels of subspecies communis (AmIb) are associated with UC cases only.^6^ The impact of *Akkermansia* on protection from IBD is controversial. In a mouse model of early-stage IBD (deletion of the transcription factor Hnf4a in intestinal epithelial cells), spikes in *Akkermansia* levels were associated with spontaneous episodes of colitis and elevated intestinal inflammation.^5^ However, the strain in these mice represented a new *Akkermansia* species, *A. ignis*.^6^ NLRP6 (NOD‐like receptor family pyrin domain containing 6) restricts *A. muciniphila* colonization. NLRP6 deficient animals are enriched for *A. muciniphila* and display higher intestinal inflammation, and IL10-/- NLRP6 -/- mice develop spontaneous colitis in a facility where IL10-/- mice do not. Furthermore, when gavaged weekly, a murine isolate of *Akkermansia* induced colitis in IL10-/- mice.^110^ However, in a different model of experimental colitis induced by Dextran Sulfate Sodium (DSS), repeated gavages with various *Akkermansia* strains, including the mouse strain “139”,^111^ MucT,^42,111,112^ and another murine isolate of *A. muciniphila*^113^ were all protective. Differences in the expansion of Tregs and lipocalin-2 levels have been reported for different *Akkermansia* strains.^111^ EVs,^112^ pasteurized *A. muciniphila* MucT, and Amuc_1100^42^ could recapitulate the effect of live *Akkermansia*. The mechanisms underlying the protection of colitis likely involve improvement of gut epithelial integrity, a boost in the number of type 3 innate immune cells,^113^ and a reduction in infiltrating macrophages and CD8+ cytotoxic T cells in the colon.^42^ In summary, *Akkermansia* levels can modulate the risk of IBD. However, specific *Akkermansia* species or strains may have no effect, be protective, or exacerbate disease depending on other variables such as the host’s genetic makeup, repeated stimulation, immune status, and diet.

#### *Diseases where* Akkermansia *abundance correlates with negative health outcomes, and disease is worsened in animal models*

Parkinson’s disease (PD) is a progressive neurodegenerative disorder characterized by the loss of dopamine-producing neurons in the brain. *Akkermansia* is increased in the microbiota of geographically distant humans with PD.^114–118^ *Akkermansia*-induced mitochondrial Ca^2+^ overload leads to the generation of reactive oxygen species and the aggregation of pathogenic αSynuclein (αSyn) in a neuroendocrine cell line. *Akkermansia* supplementation in mice induced αSyn aggregation in enteroendocrine cells, but this was not linked to more severe motor deficiency.^119^ Patients undergoing hematopoietic stem cell transplantation can develop neutropenic fevers and be at an increased risk for developing Graft vs Host Disease (GVHD). The risk for neutropenic fevers is highest in patients with an increased abundance of mucin-degrading bacteria, including *Akkermansia*. This effect can be reproduced in mice undergoing either chemical or radiation-induced immune ablation and is linked to a drastic reduction in food consumption.^120^ Under conditions of caloric restriction, some fiber-degrading bacteria turn to mucins as a nutrient source, a process exacerbated by *Akkermansia*. Indeed, administration of a murine isolate of *A. muciniphila* (strain MDA-JAX AM001) to *Akkermansia*-free mice subjected to caloric restriction led to a thinning of the mucus layer in the GI. Treatment of these animals with an antibiotic targeting *Akkermansia* preserves the mucus layer, reduces inflammation markers, and prevents hypothermia.^120^ This highlights the potential therapeutic implications of modulating *Akkermansia* abundance to mitigate the adverse effects of chemotherapy and radiotherapy in cancer patients.

Immunoglobulin A nephropathy (IgAN), a predominant cause of renal failure, is an autoimmune disorder characterized by the deposition of IgA-dominant immune complexes in the glomerular mesangium. *Akkermansia* and other mucin-degraders are increased in the microbiota of patients with IgA nephropathy.^121^ Incubation of IgA subclass 1 (IgA1) with *A. muciniphila* induces recognition by autoantibodies in the serum of these patients. Mice expressing human IgA1 and the human Fc α receptor I (α1KI-CD89tg) developed an aggravated IgA nephropathy when colonized by *A. muciniphila* MucT. *A. muciniphila* deglycosylated IgA1, which enhanced its translocation across the mouse gut to the kidney, where it forms part of immune deposits. Additionally, two risk loci for IgAN are SNPs in the alpha-defensin genes DEFA5 and DEFA6, which modulate *A. muciniphila* growth.^121^ This is an example of a combination of variance host genetics and *Akkermansia* abundance that can exacerbate the pathology of IgAN.

## Conclusions and perspectives

The accelerating number of studies linking *Akkermansia* to potential beneficial effects on humans across various disease categories presses the question of the mechanisms that enable this bacterium to impact so many aspects of human health. An emerging theme is that immunomodulation at the GI, potentially through the strengthening of gut barrier functions, can impact local and systemic inflammation. Other proposed mechanisms can contribute to overall immunomodulation, from the skewing of Treg cells^106^ to the direct secretion of TLR activators.^19,27,64^ Given that the GI is highly enervated, and most lymphocytes travel through the GI lymphatics, it is unsurprising that the microbiota and *Akkermansia* sp. would have a broader impact on immune homeostasis.

Given its potential impact as an immunomodulatory role and that viable *Akkermansia* is not always required to exert beneficial effects, this microbe presents opportunities as a therapeutic. For Type II diabetes, *Akkermansia* benefits are supported by a randomized, double-blind, placebo-controlled pilot study,^33^ with potential further investigations involving P9 and Amuc_1100. Even in this, the first and most studied disease, the impact of long-term administration of *Akkermansia* is unclear.

Animal models show evidence of *Akkermansia* intervention’s beneficial effects in several neurological diseases. Further studies must focus on determining if there are groups of patients, e.g., who harbor low *Akkermansia* levels or the “wrong” species, who would benefit from *Akkermansia* administration. Similarly, confirmatory studies in pre-clinical models and potential intervention studies in humans are needed for chronic kidney disease (CKD) and aging-related diseases like OP and atherosclerosis. In instances where protection against infection was observed, most studies were conducted in mouse models, highlighting the need for research on human subjects. In liver disease and asthma, *Akkermansia* shows promise as a protective factor in animal models, suggesting potential interventions in humans. In oncology and immunotherapies, confirmatory studies in animal models are essential to determine synergy with other therapeutics before clinical translation. In addition, new approaches are being developed to subcategorize dysbiotic states for more targeted microbial interventions. An example is the assessment of the levels of *Akkermansia* in combination with species-interacting groups (SIGs), which can correlate with overall survival in lung cancers.^39,122^

For other ailments, like IBD and other chronic GI inflammatory conditions, the impact of *Akkermansia* appears to be model-dependent and heavily influenced by diet (e.g., low-fiber content), immunosuppression, and food allergies. Mucin consumption by *Akkermansia* is predicted to negatively impact gut barrier function, particularly in the context of diets low on fiber or in low food consumption. Another limitation is that most *in vitro* studies of *Akkermansia* impact of cellular function have relied on culturing the microbe on porcine gastric mucins (Type II or III), which differ from human intestinal mucins.^1,11,12,27,38,44,47,51,119,123^ Mucin complexity also varies along the GI, with MUC5AC and MUC6 expressed in the stomach, and MUC2 the main mucin in the small intestine and colon.^124^ Colonic mucins present increased sulfation compared to gastric mucins. While *Akkermansia* can use gastric porcine O-glycans, it grows poorly on sulfated colonic mucins.^125^ Further studies involving the use of human-derived colonic mucins will aid in the elucidation of *Akkermansia* mucin metabolism in vivo.

Under some circumstances, *Akkermansia* levels are associated with increased disease severity in humans. Paradoxically, oral supplementation with *Akkermansia* has proven beneficial in conditions like SFTSV, Alzheimer’s disease, and helminth infections. Understanding the variables underlying disparate outcomes between exposure to endogenous levels of *Akkermansia* and the oral introduction of regular doses of *Akkermansia* products will help design more effective probiotic formulations.

New areas that warrant further study, considering the potential negative impact of *Akkermansia*, are its role in eating disorders and the effect of diet in *Akkermansia* interventions. Broadly, in other areas where the microbiome emerges as a modulator of health, the interaction between *Akkermansia* and other microbes should help better predict the impact of the community on the host’s physiology. Furthermore, establishing *Akkermansia* as a probiotic requires defining dosing and understanding the rules of competition between native strains and supplemented ones. The increased evidence of genetic diversity among *Akkermansia* could further refine the choice of species and strain(s) regarding therapeutic potential. The species *A. massiliensis* and *A. biwaensis* harbor higher gene content compared to *A. muciniphila*, which suggests that potentially new beneficial effects may be discovered.

Another critical area of development is determining the mechanisms of *Akkermansia* immunomodulation. For instance, while activation of TLR2 by *Akkermansia* has been proposed as responsible for strengthening epithelial tight junctions, this has not been tested formally in mice with tissue-specific TLR2 genetic ablations. Similarly, recent advances in the genetic manipulation of *Akkermansia* should allow for rigorous testing of whether proteins like Amuc_1100, P9, and Amuc_2172 have the same immunomodulatory traits in vivo as has been predicted from purified proteins.^126^ Lastly, *Akkermansia’s* impact on host physiology is likely intertwined with that of other microbiota members, as exemplified by cross-feeding interactions with *Phocaeicola vulgatus SNUG 40,005*^127^ and synergistic protective effects with *Parabacteroides* against colitis.^113^

While considerable enthusiasm surrounds the potential health benefits of *Akkermansia* (as suggested by earlier studies), the literature reviewed indicates that caution is necessary before categorizing *Akkermansia* unequivocally as a “good bug” without considering the broader context. There is a risk that some of the numerous reports of positive associations between *Akkermansia* abundance and health status have been impacted by publication bias. In this review, we highlighted the conditions and diseases associated with *Akkermansia*, differentiating between those supported by correlations alone and those including intervention studies. Additionally, we separated the findings based on studies conducted in animal models or human subjects to clarify the status of *Akkermansia* research in different areas (Figure 1). There is no prospect of implementing a “one size fits all” therapeutic approach, as the effects of *Akkermansia* depend on many factors, such as the status of the mucus barrier, GI inflammation, and especially the metabolic or neurological imbalance present in an individual. The mechanism through which *Akkermansia* would exert a beneficial versus a detrimental effect on health would be reliant on the genetics of the strain, the capacity to engraft, its mucin-utilization capabilities, the secreted proteins and metabolites, the immunomodulatory properties, the competition or cooperation with members of the host microbiota, as well as synergy with co-administered treatments.

## Data Availability

There is no research data in this paper.
